# Circulating thyroid hormones and clinical parameters of heart failure in men

**DOI:** 10.1038/s41598-023-47391-3

**Published:** 2023-11-20

**Authors:** Iva Turić, Ivan Velat, Željko Bušić, Viktor Čulić

**Affiliations:** 1 Department of Cardiology and Angiology, University Hospital Centre Split, 21000 Split, Croatia; 2 Department of Urology, University Hospital Centre Split, Split, Croatia; 3 Department of Neurosurgery, University Hospital Centre Split, Split, Croatia; 4 University of Split School of Medicine, Split, Croatia

**Keywords:** Cardiology, Endocrinology

## Abstract

Heart failure (HF) is a multiple hormonal deficiency syndrome which includes alterations in the serum concentration of thyroid hormones (TH). This cross-sectional study enrolled 215 male patients hospitalised for acute HF. Data on cardiovascular risk factors, chronic medications, cardiac function assessed by echocardiography, and clinical parameters of HF were prospectively collected. The independent predictive association of TH with all investigated parameters of the HF severity were assessed. The patient’s mean age was 74.4 years, 57.2% had arterial hypertension, 54.0% were consuming alcohol, and 42.3% were diabetics. Multivariate analysis revealed that total triiodothyronine (TT_3_) was an independent predictor of greater left ventricular ejection fraction (LVEF; *β* = 0.223, *p* = 0.008), less progressed left ventricular diastolic dysfunction (LVDD; *β* = − 0.271, *p* = 0.001) and lower N-terminal pro-brain natriuretic peptide (NT-proBNP; *β* = − 0.365, *p* < 0.001). None of the TH other than TT_3_ was associated with LVDD or NT-proBNP, whereas free triiodothyronine (*β* = − 0.197, *p* = 0.004), free thyroxine (*β* = − 0.223, *p* = 0.001) and total thyroxine (*β* = − 0.140, *p* = 0.041) were inversely associated with LVEF. The present study suggests that, among TH, serum TT_3_ level is most closely associated with echocardiographic, laboratory and clinical parameters of the severity of HF in men.

## Introduction

Heart failure (HF), a common final pathway of numerous cardiac disorders, is characterised by a complex pathophysiology where hormonal imbalance plays an important role^1, 2^. This multiple hormonal deficiency syndrome includes lower circulating levels of growth hormone, insulin-like growth factor-1, dehydroepiandrosterone sulphate, and testosterone^3–6^. Alterations in the serum concentration of thyroid hormones (TH) have also been associated with HF.

The non-thyroidal illness syndrome (NTIS) is a set of changes in the circulating concentrations of TH commonly seen in advanced HF. NTIS is represented by a decrease in serum triiodothyronine (T_3_) levels which is, in more severe and prolonged cases of the syndrome, associated with a reduction in serum thyroxine (T_4_). These changes are not followed by an expected increase in the serum concentrations of thyroid-stimulating hormone (TSH)^7^. It has been suggested that between 18 and 30% of patients with congestive HF develop a drop in serum levels of T_3_^8–11^. This drop has been associated with a reduced left ventricular ejection fraction (LVEF), a higher degree of diastolic dysfunction of the left ventricle (LVDD), and an increased risk of death^12–14^. All these cardiac abnormalities may be reversible with the restoration of normal thyroid hormone values^15^.

There are important differences between men and women in almost all aspects of HF due to gender-specific factors, primarily involving sex hormones^16^. Testosterone, the primary sex hormone in men, has been associated with LVDD and clinical parameters of HF in male patients^17–20^. The peripheral effects of testosterone may explain most of its favourable effects on the pathophysiology of HF, but its central effects need further exploration^21^.

So far, the relationship between TH and the parameters of HF has not been investigated in greater detail in male patients with HF. The present study aims to investigate the association of five TH, namely free and total T_3_ (fT_3_ and TT_3_), free and total T_4_ (fT_4_ and TT_4_), and TSH, with LVEF and LVDD, and other clinical parameters of HF severity in men, with controlling for the effect of serum testosterone levels and other clinical factors.

## Methods

This cross-sectional study enrolled male patients admitted for acute HF at the Department of Cardiology and Angiology, University Hospital Centre Split, between February 2016 and October 2019. Data on baseline characteristics, the existence of cardiovascular risk factors, cardiovascular drugs from the patient's chronic therapy, blood determinations, and echocardiographic parameters were prospectively collected during the patient's hospital stay. The participant’s height and weight, wearing hospital clothes without shoes, were measured during the clinical examination at the beginning of the hospitalization, and body mass index (BMI, in kg/m^2^) was calculated. The Ethics Committee from the Science Department of the University Hospital Centre Split approved the study protocol, and each participant gave his written informed consent (2181-147-10-01/01-M. J). The study complies with the Helsinki Declaration.

Inclusion criteria were: (1) clinical presentation typical for HF; (2) LVEF equal to or less than 50% (using the Simpson method), and/or LVDD established by transthoracic echocardiography; (3) unchanged medical therapy during at least one month. Exclusion criteria were: (1) acute or chronic systemic illness that could affect hormonal metabolism (i.e., a primary endocrine disorder, autoimmune or malignant disease, infection, terminal phase of renal failure, primary liver disease and liver cirrhosis), including patients with the BMI less than 18.5 kg/m^2^ for potential frailty; (2) any hormonal treatment or drugs significantly influencing TH levels at the time of the study or in the past (i.e., antithyroid drugs, synthetic TH, corticosteroids, dopamine, dobutamine); (3) cardiac surgery, acute coronary syndrome or coronary revascularization within six months before the study; (4) C-reactive protein levels above 15 mg/dL. C-reactive protein levels under 10 mg/L may be interpreted as of minor clinical importance^22^ and considering a low-grade systemic inflammation that accompanies HF^23^, we established a maximum of 15 mmol/L, i.e., a 50% increase, as a cut-off value for inclusion^24^.

Blood samples for blood count and biochemical analysis were taken on admission, (i.e., during the patient’s evaluation in the emergency department), from the anterior cubital vein. Circulating TH and total testosterone levels were assessed within the first three days of hospitalization using commercially available radioimmunoassay kits (Roche Diagnostics GmbH, Mannheim, Germany). The inter-assay coefficient for all hormones ranged between 8 and 13%. For all patients, these blood samples were taken between 8 and 9 a.m. to account for circadian variation. According to the manufacturer's instructions in our laboratory, normal reference intervals were 1.34 to 2.73 nmol/L for TT_3_, 3.8–6.0 pmol/L for fT_3_, 78–157 nmol/L for TT_4_, 7.90–14.40 pmol/L for fT_4_, and 0.34–5.60 mIU/L for TSH. The estimated glomerular filtration rate (eGFR, in mL/min/1.73 m^2^) was calculated using the Modification of Diet in Renal Disease equation^25^.

Transthoracic echocardiography was performed by certified cardiologists using a Vivid 9E device (GE Medical System, Milwaukee WI, USA) and interpreted according to the standard guidelines of the European Society of Cardiology (ESC) for LVEF^26^ and LVDD assessment^27^. The LVEF was calculated using the Simpson biplane method. The LVDD was assessed by measuring the mitral valve inflow on pulsed-wave Doppler in the four-chamber view (E and A velocity waves, E/A ratio, E-wave deceleration time, isovolumic relaxation time) and septal and lateral peak annular tissue velocities (é and á waves, the é/á ratio, E/é ratio) obtained by tissue-Doppler and pulsed-wave Doppler imaging. The pulsed-wave Doppler sample volumes were positioned within one centimetre of the septal and lateral insertion of the mitral valve leaflets. According to LVEF, we stratified our patients into two categories, normal, with LVEF greater than 50%, and reduced, with LVEF equal to or less than 50%. LVDD was assessed according to the ESC recommendations and divided into three basic grades^28^. However, to provide a more detailed assessment, we divided the restrictive pattern of mitral inflow into reversible and irreversible types and included intermediate grades where appropriate^17^.

Data were expressed as percentages for dichotomous variables, as mean value ± standard deviation (SD) for normally distributed continuous variables, and through medians and interquartile ranges (IQR) for nonuniformly distributed continuous variables. The normality of data distribution was tested using the Kolmogorov–Smirnov test. In the univariate analysis, differences between the groups were tested using the *χ*^2^ test, *t*-test, or Mann–Whitney *U* test, as appropriate. Multiple regression analysis was used to assess the independent predictive association of the TH, total testosterone levels, age, BMI, eGFR, traditional risk factors, previous myocardial infarction (MI), and cardiovascular medications with the echocardiographic parameters (LVEF and LVDD), N-terminal pro-brain natriuretic peptide (NT-proBNP), New York Heart Association (NYHA) functional class and HF duration. These results were expressed through *β* and *p*-values. The statistical significance of the test was determined by a *p*-value less than 0.05. Data were analysed with IBM SPSS Statistics 26 (26.0.0.0; 2019; Armonk, New York; USA).

## Results

### Characteristics of the study population

Tables 1 and 2 display the baseline characteristics, prehospital medication, and laboratory findings of the 215 included men. The median duration of HF was 24.0 months, and the patient’s mean age was 74.4 years. The average LVEF was 46.1%; 41.4% of the included patients had preserved LVEF, 23.7% had mid-range LVEF, and 34.9% had reduced LVEF. All patients had LVDD with an average grade of 2.7 ± 0.6. More than half of the patients had arterial hypertension or were currently consuming alcohol, while 42.3% of them were diabetics. Approximately two-thirds of the patients used loop diuretics in chronic therapy.

**Table 1:** Baseline characteristics and prehospital medication of the study patients.

	All patients *n* = 215
Baseline characteristics	
Age (mean ± SD; years)	74.4 ± 8.0
Body mass index (mean ± SD)	27.5 ± 5.1
NYHA class (mean ± SD)	3.4 ± 0.6
HF duration (median, IQR; months)	24.0 (5.0–116.0)
Arterial hypertension (*n*, %)	123 (57.2)
Diabetes mellitus (*n*, %)	91 (42.3)
Hyperlipidaemia (*n*, %)	59 (27.4)
Previous MI (*n*, %)	44 (20.5)
Smoking (*n*, %)	27 (12.6)
Alcohol consumption (*n*, %)	116 (54.0)
Prehospital medication (*n*, %)	
Loop diuretic	142 (66.0)
Spironolactone	47 (21.9)
Hygroton	6 (2.8)
Beta-blocker	114 (53.0)
Calcium channel blocker	34 (15.8)
ACEI	88 (40.9)
ARB	28 (13.0)
Aspirin	57 (26.5)
Clopidogrel	27 (12.6)
Nitrate	13 (6.0)
Trimetazidine	16 (7.4)
Digoxin	49 (22.8)
Statin	48 (22.3)
Amiodarone	7 (3.3)

*SD* standard deviation, *NYHA* New York Heart Association, *HF* heart failure, *MI* myocardial infarction, *ACEI* angiotensin-converting enzyme inhibitor, *ARB* angiotensin II-receptor blocker.

**Table 2:** Laboratory findings of the study patients.

Laboratory findings*	All patients *n* = 215
Haemoglobin level (g/L)	129.1 ± 22.0
Haematocrit (L/L)	0.4 ± 0.1
Serum urea (median, IQR; mmol/L)	8.2 (6.1–11.9)
Serum creatinine (median, IQR; µmol/L)	1.4 (1.1–1.8)
Glomerular filtration rate (mL/min/1.73 m^2^)	55.8 ± 19.5
Urates (mg/dL)	496.9 ± 139.0
High-sensitive troponin (median, IQR; ng/mL)	0.1 (0.0–0.2)
NT-proBNP (median, IQR; pmol/L)	479.6 (182.3–1442.9)
Total testosterone (median, IQR; nmol/L)	10.2 (6.0–14.0)
Direct bilirubin (median, IQR; µmol/L)	4.6 (3.1–7.2)
Indirect bilirubin (median, IQR; µmol/L)	13.8 (9.1–20.0)
AST (median, IQR; U/L)	25.0 (20.0–34.0)
ALT (median, IQR; U/L)	24.0 (18.0–35.0)
GGT (median, IQR; U/L)	58.0 (27.0–110.0)
Alkaline phosphatase (median, IQR; U/L)	74.0 (57.0–100.0)
Total proteins (g/L)	70.3 ± 8.6
Serum albumins (g/L)	37.4 ± 4.4
Serum globulins (g/L)	32.2 ± 7.6
Serum sodium (mmol/L)	138.1 ± 4.8
Serum potassium (mmol/L)	4.3 ± 0.6
Serum chloride (mmol/L)	99.0 ± 5.8
Serum calcium (mmol/L)	2.4 ± 0.2
Serum magnesium (mg/dL)	0.8 ± 0.1

*Values are represented as mean ± standard deviation unless otherwise noted (median, 25th–75th percentile).

*IQR* interquartile range, *NT-proBNP* N-terminal pro-brain natriuretic peptide, *AST* aspartate aminotransferase, *ALT* alanine aminotransferase, *GGT* gamma-glutamyl transferase, *LDL* low-density lipoprotein, *HDL* high-density lipoprotein.

The analysis of interrelations among TH revealed that there were significant positive correlations among fT_3_ and both fractions of T_4_ as well as among fT_4_, TT_3_ and TT_4_. In addition, there was a borderline positive correlation between fT_3_ and TT_3_ and a significant inverse correlation between TSH and both fT_4_ and TT_4_ (Supplementary Table S1).

### TT_3_ and parameters of HF

**Figure 1:**
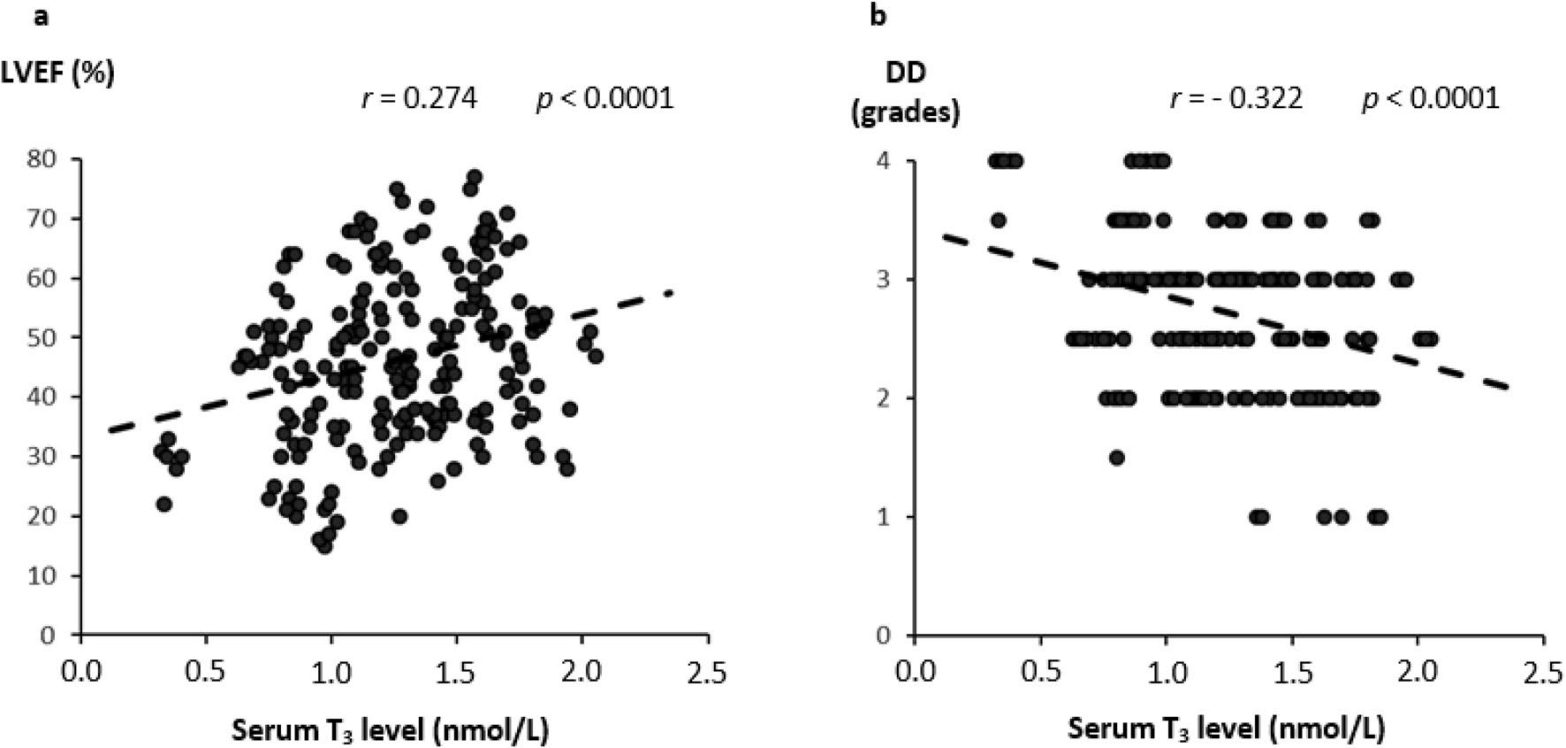
Correlation between serum total triiodothyronine (T_3_) level and the echocardiographic parameters of cardiac function. Scatterplots depicting the correlation between serum total triiodothyronine (T_3_) level and the echocardiographic parameters of cardiac systolic and diastolic function in the study population. Significant correlations were observed for both the left ventricular ejection fraction (LVEF; panel (**a**), linear regression equation: Y = 10.34X + 33.14) and the grade of diastolic dysfunction (DD; panel (**b**), linear regression equation: Y = − 0.57X + 3.43).

**Figure 2:**
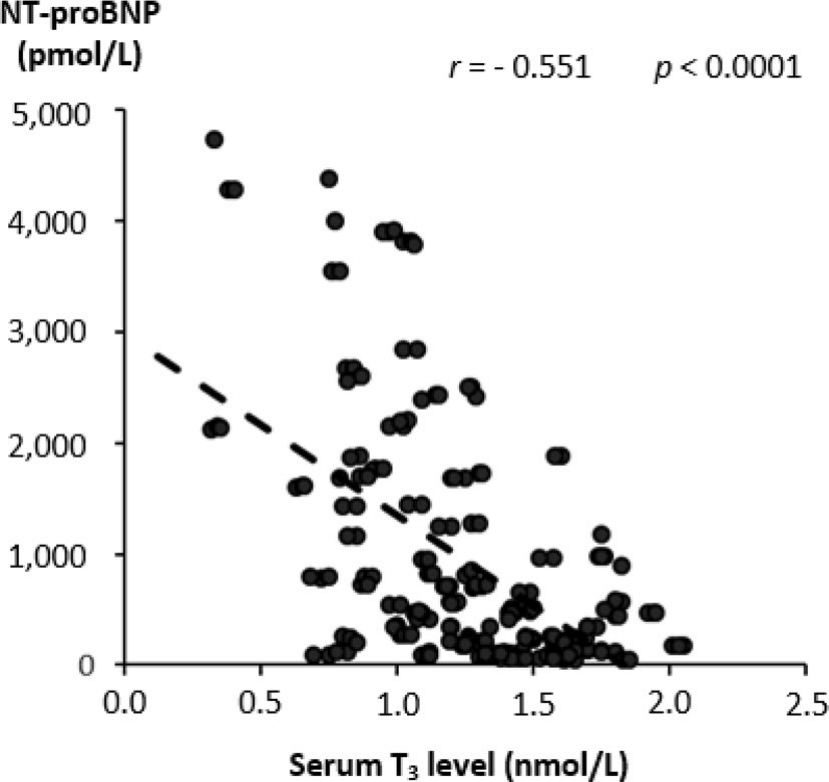
Correlation between serum total triiodothyronine (T_3_) level and N-terminal pro-brain natriuretic peptide (NT-proBNP) level. Scatterplot depicting the correlation between serum total triiodothyronine (T_3_) level and serum N-terminal pro-brain natriuretic peptide (NT-proBNP) level in the study population. Linear regression equation: Y = − 1628.1X + 2980.1. Regression equation, *r* and *p-*values were obtained from the linear regression analysis.

In the univariate analysis, serum TT_3_ levels were significantly associated with the echocardiographic parameters of cardiac function showing a positive correlation with LVEF and an inverse correlation with LVDD (*p* < 0.0001; Fig. 1). TT_3_ level also inversely correlated with NT-proBNP level (*p* < 0.0001; Fig. 2) and NYHA class (*r* = − 0.207, *p* = 0.002), whereas there was no significant relationship with the duration of HF (*r* = − 0.47, *p* = 0.56). Multivariate analysis confirmed that all the aforementioned associations, except for NYHA class and HF duration, are independent of other clinical factors, including serum testosterone levels (Tables 3, 4, 5, Supplementary Tables S2 and S3).

**Table 3:** Multiple regression analysis for the predicting association of TT_3_ levels and other clinical factors with left ventricular ejection fraction.

	*β*^†^	*p*^‡^
Predictors		
Age (years)	0.118	0.130
BMI (kg/m^2^)	− 0.069	0.374
Total T_3_	0.223	0.008*
Total testosterone (nmol/L)	0.201	0.006*
Glomerular filtration rate (mL/min/1.73 m^2^)	0.134	0.104
Arterial hypertension	0.049	0.591
Diabetes mellitus	− 0.136	0.076
Hyperlipidaemia	0.131	0.057
Previous MI	− 0.009	0.903
Smoking	0.021	0.761
Alcohol consumption	0.002	0.981
Loop diuretic	0.144	0.083
Spironolactone	− 0.099	0.174
Beta-blocker	− 0.113	0.156
Calcium channel blocker	0.191	0.011*
ACEI	0.072	0.314
ARB	0.034	0.648
Digoxin	− 0.056	0.449

*Statistically significant (*p* < 0.05).

^†,‡^*β* and *p-*values were obtained from the multiple regression analysis.

*TT*_*3*_ total triiodothyronine, *HF* heart failure, *BMI* body mass index, *MI* myocardial infarction, *ACEI* angiotensin-converting enzyme inhibitor, *ARB* angiotensin II-receptor blocker.

**Table 4:** Multiple regression analysis for the predicting association of TT_3_ levels and other clinical factors with left ventricular diastolic dysfunction.

	*β*^†^	*p*^‡^
Predictors		
Age (years)	0.104	0.173
BMI (kg/m^2^)	0.149	0.052
Total T_3_	− 0.271	0.001*
Total testosterone (nmol/L)	− 0.214	0.003*
Glomerular filtration rate (mL/min/1.73 m^2^)	− 0.054	0.507
Arterial hypertension	0.023	0.800
Diabetes mellitus	0.118	0.116
Hyperlipidaemia	− 0.064	0.342
Previous MI	− 0.074	0.279
Smoking	0.034	0.610
Alcohol consumption	0.062	0.367
Loop diuretic	− 0.223	0.007*
Spironolactone	0.114	0.113
Beta-blocker	− 0.029	0.713
Calcium channel blocker	− 0.236	0.001*
ACEI	0.214	0.003*
ARB	0.122	0.091
Digoxin	0.041	0.575

*Statistically significant (*p* < 0.05).

^†,‡^*β* and *p-*values were obtained from the multiple regression analysis.

*TT*_*3*_ total triiodothyronine, *HF* heart failure, *BMI* body mass index, *MI* myocardial infarction, *ACEI* angiotensin-converting enzyme inhibitor, *ARB* angiotensin II-receptor blocker.

**Table 5:** Multiple regression analysis for the predicting association of TT_3_ levels and other clinical factors with NT-proBNP levels.

	*β*^†^	*p*^‡^
Predictors		
Age (years)	− 0.083	0.126
BMI (kg/m^2^)	0.025	0.648
Total T_3_	− 0.365	< 0.001*
Total testosterone (nmol/L)	− 0.234	< 0.001*
Glomerular filtration rate (mL/min/1.73 m^2^)	− 0.428	< 0.001*
Arterial hypertension	− 0.005	0.939
Diabetes mellitus	− 0.049	0.358
Hyperlipidaemia	0.056	0.238
Previous MI	− 0.090	0.067
Smoking	− 0.029	0.538
Alcohol consumption	0.076	0.119
Loop diuretic	0.053	0.356
Spironolactone	0.164	0.001*
Beta-blocker	0.123	0.027*
Calcium channel blocker	− 0.078	0.130
ACEI	− 0.015	0.765
ARB	− 0.232	< 0.001*
Digoxin	− 0.010	0.844

*Statistically significant (*p* < 0.05).

^†,‡^*β* and *p-*values were obtained from the multiple regression analysis.

*TT*_*3*_ total triiodothyronine, *NT-proBNP* N-terminal pro-brain natriuretic peptide, *HF* heart failure, *BMI* body mass index, *MI* myocardial infarction, *ACEI* angiotensin-converting enzyme inhibitor, *ARB* angiotensin II-receptor blocker.

### Other TH and parameters of HF

Univariate analysis showed that TSH, fT_3_ and fT_4_ were inversely related to LVEF, while none of the TH other than TT_3_ was associated with LVDD (Supplementary Table S4). A positive correlation was observed between TSH and both NT-proBNP and NYHA class, while there was an inverse correlation between TSH and HF duration (Supplementary Table S4). Multivariate analysis confirmed the independent association only between TSH and HF duration (Supplementary Table S5) whereas there was no independent association between TSH and LVEF, LVDD, NT-proBNP or NYHA class (Supplementary Tables S6–S9). Independent predictors of LVEF were lower levels of fT_3_ (Supplementary Table S10), fT_4_ (Supplementary Table S11) and TT_4_ (Supplementary Table S12). Also, after adjustment for other clinical variables, fT_3_, fT_4_, and TT_4_ were not associated with LVDD (Supplementary Tables S13, S4 and S15) or NT-proBNP (Supplementary Tables S16, S17 and S18). There were independent associations of fT_4_ with HF duration (Supplementary Table S19) and TT_4_ with NYHA class (Supplementary Table S20) whereas there were no predictive associations between fT_3_, fT_4_, and TT_4_ and the rest of the observed parameters (Supplementary Tables S21–S24).

### Other variables and parameters of HF

In all multivariate analyses, total testosterone also showed substantial associations with LVEF (Table 3, Supplementary Tables S6, S10–S12), LVDD (Table 4, Supplementary Tables S7, S13–S15) and NT-proBNP (Table 5, Supplementary Tables S8, S16–S18); those associations were of similar nature and strength as the associations observed for TT_3_. Age and non-use of alcohol were independently associated with HF duration in all multiple regression analyses (Supplementary Tables S3, S5, S19, S22 and S24).

## Discussion

Our study showed that, among the five investigated TH, serum TT_3_ levels had the closest correlation with echocardiographic, laboratory and clinical parameters of the HF severity in men. These associations were independent of other clinical variables and factors, including serum testosterone levels.

TH are important modulators of cardiac function through cardiac and extra-cardiac mechanisms. They influence the heart rate, heart rhythm, myocardial contraction, and blood pressure, and have an impact on cardiovascular risk factors, such as hyperlipidaemia, arterial hypertension and thrombogenesis^15^. The main alteration of TH in HF, the NTIS, is characterised by changes in the TH pathophysiology both at the level of the hypothalamic–pituitary–thyroid axis and at the organ and tissue level^7^. In the acute phase of a critical illness, concomitant with a drop in serum T_3_ level, a transient rise in serum T_4_ level may be seen^29^. The disruption of the thyroid axis occurs when the illness is prolonged, which may result in a decrease in serum T_4_ and TSH levels^29^. Changes in TH levels that accompany NTIS may have prognostic significance for the worsening of HF and may represent independent predictors of cardiac and overall mortality^12, 30–33^.

The T_3_ tissue availability is regulated through the expression of TH membrane transporters (monocarboxylate transporters 8 and 10, and organic anion transporting polypeptide family 1C1), iodothyronine deiodinases (DIO1, DIO2 and DIO3), intracellular thyroid receptors (α1, β1 and β2) and hypothalamus thyrotropin-releasing hormone (TRH)^7, 34–36^. In NTIS, it has been reported that factors such as systemic inflammation^36^, glucocorticoid serum rise^37^ or energy status^38^ may alter the expression of the above-mentioned regulators, resulting in the T_3_ serum and tissue reduction. Experimental studies have shown that proinflammatory cytokines (interleukins 6, 1 and 1β, and tumour necrosis factor α) downregulate various components of TH synthesis and metabolism^39, 40^, whereas high glucocorticoid levels are responsible for suppressing the pituitary response to TRH in men^37, 41^. During food deprivation, as an example of low energy status, a drop in serum leptin is present, which induces a decrease in TRH expression in the hypothalamus^42^. Different components of TH level regulation are altered differently depending on the type of tissue, duration, severity, and type of the illness^7, 43^. However, regardless of the important advances, there are multiple gaps in the current knowledge of the pathophysiology of NTIS.

Selvaraj et al. have reported that in HF with preserved LVEF, serum TT_3_ has been negatively associated with NT-proBNP and severe LVDD^44^. In patients suffering from idiopathic LV dysfunction, TT_3_ has been suggested as the predictor of NYHA class only, without association with either LVEF or NT-proBNP^45^. In neonatal rat ventricular myocytes, exposure to T_3_ increased gene expression of brain natriuretic peptide (BNP)^46^. However, it has also been reported that BNP gene expression is generally increased during the fetal period, as an important regulator of myocyte growth^47^. The expression of BNP genes and other genes typically expressed during the fetal period is enhanced during HF^48, 49^. In a recent study, adult rats^49^, one group with propylthiouracil-induced hypothyroidism and another one with MI-induced HF, have been investigated. The results have suggested that the BNP expression is negatively regulated by cardiac tissue T_3_ levels following oral T_3_ treatment in both groups. In addition, the increasing serum TT_3_ showed a strong inverse correlation with serum BNP, which is in accordance with our results. Therefore, both that^49^ and our study suggest that BNP could serve as a serum biomarker of cardiac tissue T_3_ activity.

For the first time, our results revealed strong predictive associations of TT_3_ and all three important parameters of HF severity, namely LVEF, LVDD and NT-proBNP. It is important to stress that all of these associations are independent of the effects of other relevant clinical variables, including testosterone levels. Our study strongly supports the role of TT_3_ as the chief TH involved in the modulation of cardiac function in terms of HF mechanisms and progression. This is in line with the principal role of TT_3_ in preserving LVEF^50^, probably through the favourable influence of T_3_ on cardiac contractility and reduction of myocardial damage^15, 51–55^. The beneficial role on LVDD may also be explained by the reduction of myocardial fibrosis^56–58^ through cardioprotective mechanisms of T_3_, such as activation of cytoprotective mechanisms, metabolic adaptation and neoangiogenesis^15^.

In addition, T_3_ has been implicated in the reduction of peripheral vascular resistance^59, 60^. An increase in peripheral vascular resistance has been proposed as a contributor to HF progression through deleterious and irreversible effects on the heart and circulation^61^. The combined effect of all of the aforementioned mechanisms may be responsible for the overall beneficial effect of TT_3_ in HF, expressed through a strong inverse relation with NT-proBNP in our patients.

Despite the significant impact of T_3_ on cardiac function, previous data have shown no clear association of fT_3_ with the parameters of HF severity. One previous study has reported a positive relationship of fT_3_ with NT-proBNP levels^32^, whereas several other studies have reported no relationship with LVEF^33, 62^. However, none of these associations have been adjusted for important clinical confounders. Experimental data on intravenous T_3_ therapy have shown that fT_3_ levels, raised by infusion, are associated with an improved stroke volume and end-diastolic LV volume, lower NT-proBNP levels and signs of deactivation of neuroendocrine mechanisms^63^.

Our multivariate analysis revealed an inverse relationship between fT_3_ and LVEF, whereas there was no independent relationship between fT_3_ and LVDD or NT-proBNP. In the context of the above-described data^63^, this may represent a compensatory mechanism of enhanced increase of fT_3_ aiming to improve LVEF.

The evidence on the relationship of TT_4_ and fT_4_ with cardiac function parameters is scarce. In patients with dilated cardiomyopathy, a positive relationship with NT-proBNP and no relationship with the grade of LVDD has been reported for fT_4_^44^. Randomised controlled studies on relatively small populations of patients with dilated cardiomyopathy have demonstrated an improvement in LVEF and parameters of longitudinal and circumferential strain after receiving substitutional therapy with levothyroxine, the synthetic form of T_4_^64–66^.

Our results showed a negative association of TT_4_ and fT_4_ with LVEF and no independent association with LVDD and NT-proBNP. Also, TT_4_ was a predictor of NYHA class, whereas fT_4_ was a predictor of HF duration. During NTIS, the conversion of T_4_ to T_3_ is reduced^7^. In general, TH nuclear receptors bind T_3_ with a 10 times greater affinity than T_4_^67^. Accordingly, it has been suggested that cardiac TH-responsive genes are expressed as a function of serum T_3_, whereas, regardless of its serum levels, T_4_ may have a lower impact on cardiac function^15^. The fact that we observed an inverse association of TT_4_ and fT_4_ with TSH may suggest that this part of the thyroid feedback loop is less disrupted by the pathological mechanisms of HF. The observed associations may also be interpreted as a compensatory rise of TT_4_ and fT_4_ in patients with more severe HF, as in the case of fT_3_. In this context, higher T_4_ levels combined with lower T_3_ levels may indicate a lower peripheral conversion of TH^45, 68^. Since both T_4_ and T_3_ substitutional therapy have been related to the improvement of cardiac function in several studies^63–66^, the T_3_/T_4_ ratio could be useful in monitoring future therapy options.

In patients with dilated cardiomyopathy, TSH level has been associated with NT-proBNP levels^44^, whereas no significant relationship between TSH and echocardiographic parameters of cardiac size and function has been observed^62^. In patients with subclinical hypothyroidism, significant negative correlations of TSH with cardiac index, stroke volume and end-diastolic volume, as well as a positive relationship of TSH with systemic vascular resistance have been described^69^. However, for all of the reported relationships, no adjustments were made for relevant clinical variables.

TRH and TSH levels are regulated by a typical endocrine feedback loop^70^. Nevertheless, there is no compensatory rise in serum TSH in NTIS following a reduction in serum levels of other TH^7^. It has been suggested that factors such as age, sex, body mass index, race, smoking habits, iodine intake, the timing of the sample acquisition, as well as accompanying medical conditions and medications can affect serum TSH levels^15^. In our study, after adjusting for total testosterone levels, age, BMI, eGFR, traditional cardiovascular disease risk factors, previous MI and prehospital cardiovascular medications, no association between TSH and LVEF, LVDD and NT-proBNP was found. Therefore, our study suggests that serum TSH levels are not helpful in the assessment of HF progression and are not associated with cardiac function parameters.

It has been hypothesised that the beneficial effect of testosterone may be exerted only on a relatively healthy heart unaffected by pathological remodelling^17,71^. Total testosterone has been suggested as an independent predictor of NT-proBNP, LVDD and NYHA class^24, 72, 73^. The present study confirmed that the associations of testosterone with HF parameters are independent of TH.

HF is a long-term syndrome that has a more severe form in older patients^74^. Excessive consumption of alcohol has been related to alcoholic cardiomyopathy^75, 76^. In contrast, the beneficial effects of light-to-moderate alcohol consumption in HF have been well-established^77–80^. In our study, the average age of the patients was 74.4 years with a median duration of HF of 24.0 months. Age and non-use of alcohol were independent predictors of HF duration in all adjusted analyses. We may only speculate that younger patients may have had more rapidly progressed forms of HF and died sooner. In the same manner, shorter HF duration in patients consuming alcohol could suggest predominantly adverse effects of alcohol associated with a worse overall prognosis.

The main strength of our study is the extensive exclusion criteria covering conditions that could have had an impact on the variables of interest. Moreover, in contrast to the majority of previous clinical studies, in the present study, we adjusted the associations of TH with HF parameters for a number of important clinical confounders.

Since we investigated a highly selected population, created to obtain as representative sample as possible, our sample was not suitable for the assessment of specific roles of traditional cardiovascular risk factors and cardiovascular medications. Additionally, we did not record the exact doses of the medicines in chronic therapy, nor did we detail the amounts of alcohol consumption or the iodine intake. We measured serum levels of TH, whereas the exact cardiac tissue TH levels remained uncertain. We did not collect data on the aetiology of HF. Furthermore, there is a possibility of a measurement bias considering that the echocardiography exams were not performed by the same person.

Despite the possible impact of T_3_ on cardiac function and the experimental data showing an improvement in cardiac function following a rise in fT_3_ serum levels, we observed a discrepancy between fT_3_ and TT_3_ in their correlation with HF parameters. We may assume that multiple clinical factors in the real-world setting coupled with the complex TH metabolism could have underlined these observations. Another limitation of the present study is its cross-sectional nature, which prevents observing the relationship between cause and effect. Since we only included male HF patients, whether the results can be generalised to female HF patients and other patient populations is questionable.

At the moment, there are no recommendations for the type and dose of the TH substitutional therapy, nor for the monitoring of the therapy effect in patients with NTIS. For example, inconclusive results have been obtained for the efficacy of the T_4_ + T_3_ combination as a treatment option in clinical hypothyroidism and for restoring euthyroidism^81, 82^. This therapy has been suggested by the European Thyroid Association for patients with persistent complaints despite previous substitutional levothyroxine therapy and serum TSH values within the reference range, provided they have received the needed psychological support and have no autoimmune disorders^83^. As we observed that all investigated TH, except for TSH, were associated with LVEF, there is a possibility that T_4_ + T_3_ therapy, through the complex pathways of metabolic effects of TH, may be useful for patients with clinical HF. Additional experimental and clinical studies are needed to explore whether this or some other treatment option may produce clinically meaningful improvement of TT_3_ associated with an improvement in the clinical course, symptom severity and functional status of HF patients.

## Conclusion

There is still a considerable lack of knowledge about the role of TH in the pathophysiology and prognosis of patients with HF. Our study suggests that among TH, serum TT_3_ level is most closely associated with echocardiographic, laboratory and clinical parameters of the severity of HF in men, independently of other clinical variables and factors, including circulating testosterone levels.

### Supplementary Information

Supplementary Tables.

## Data Availability

The dataset analysed in the present study is available from the corresponding author upon reasonable request.
