# Supplementary material for: Circulating thyroid hormones and clinical parameters of heart failure in men
Source: Sci Rep. 2023 Nov 20;13:20319. doi: 10.1038/s41598-023-47391-3 (PMC10662258; doi:10.1038/s41598-023-47391-3)
Supplement: Supplementary file 1 — Supplementary Tables. [file 41598_2023_47391_MOESM1_ESM.pdf]

Supplementary material

Circulating thyroid hormones and clinical parameters of heart failure in men

Iva Turić, MD, Ivan Velat, MD, Željko Bušić, MD, Viktor Čulić, MD, PhD

Table of contents

Supplementary Table S1...... Correlations between thyroid hormones**..1**
Supplementary Table S2...... Multiple regression analysis for the predicting association of TT_3 levels and other clinical factors with NYHA class**..2**
Supplementary Table S3...... Multiple regression analysis for the predicting association of TT_3 levels and other clinical factors with HF duration**...3**
Supplementary Table S4...... Correlation between echocardiographic parameters of cardiac function, heart failure clinical parameters, and thyroid hormones other than TT_3**..4**
Supplementary Table S5...... Multiple regression analysis for the predicting association of TSH levels and other clinical factors with HF duration**...5**
Supplementary Table S6...... Multiple regression analysis for the predicting association of TSH levels and other clinical factors with left ventricular ejection fraction**..6**
Supplementary Table S7...... Multiple regression analysis for the predicting association of TSH levels and other clinical factors with left ventricular diastolic dysfunction**..7**
Supplementary Table S8...... Multiple regression analysis for the predicting association of TSH levels and other clinical factors with NT-proBNP levels**...8**
Supplementary Table S9...... Multiple regression analysis for the predicting association of TSH levels and other clinical factors with NYHA class**..9**
Supplementary Table S10...... Multiple regression analysis for the predicting association of fT_3 levels and other clinical factors with left ventricular ejection fraction**..10**
Supplementary Table S11...... Multiple regression analysis for the predicting association of fT_4 levels and other clinical factors with left ventricular ejection fraction**..11**
Supplementary Table S12...... Multiple regression analysis for the predicting association of TT_4 levels and other clinical factors with left ventricular ejection fraction**..12**
Supplementary Table S13...... Multiple regression analysis for the predicting association of fT_3 levels and other clinical factors with left ventricular diastolic dysfunction**...13**

Supplementary Table S14...... Multiple regression analysis for the predicting association of fT_4 levels and other clinical factors with left ventricular diastolic dysfunction**..14**
Supplementary Table S15...... Multiple regression analysis for the predicting association of TT_4 levels and other clinical factors with left ventricular diastolic dysfunction**..15**
Supplementary Table S16...... Multiple regression analysis for the predicting association of fT_3 levels and other clinical factors with NT-proBNP levels**..16**
Supplementary Table S17...... Multiple regression analysis for the predicting association of fT_4 levels and other clinical factors with NT-proBNP levels**..17**
Supplementary Table S18...... Multiple regression analysis for the predicting association of TT_4 levels and other clinical factors with NT-proBNP levels**..18**
Supplementary Table S19...... Multiple regression analysis for the predicting association of fT_4 levels and other clinical factors with HF duration**..19**
Supplementary Table S20...... Multiple regression analysis for the predicting association of TT_4 levels and other clinical factors with NYHA class**..20**
Supplementary Table S21...... Multiple regression analysis for the predicting association of fT_3 levels and other clinical factors with NYHA class**..21**
Supplementary Table S22...... Multiple regression analysis for the predicting association of fT_3 levels and other clinical factors with HF duration**..22**
Supplementary Table S23...... Multiple regression analysis for the predicting association of fT_4 levels and other clinical factors with NYHA class**..23**
Supplementary Table S24...... Multiple regression analysis for the predicting association of TT_4 levels and other clinical factors with HF duration**..24**

Supplementary Table S1. Correlations between thyroid hormones.

	TT_3 [nmol/L]		fT_3 [pmol/L]		fT_4 [pmol/L]		TT_4 [nmol/L]	
	r †	*p*	*r*	*p*	*r*	*p*	*r*	*p*
fT_3 [pmol/L]	0.134	0.050						
fT_4 [pmol/L]	0.162	0.017*	0.374	<0.001*				
TT_4 [nmol/L]	0.266	<0.001*	0.598	<0.001*	0.625	<0.001*		
TSH [mIU/L]	-0.093	0.174	-0.069	0.316	-0.202	<0.001*	-0.176	0.010*

* Statistically significant ($p<0.05$).

† Pearson's coefficient.

The normality of data distribution was tested using the Kolmogorov-Smirnov test.

TT_3: total triiodothyronine, fT_3: free triiodothyronine, fT_4: free thyroxine, TT_4: total thyroxine, TSH: thyroid stimulating hormone.

Supplementary Table S2. Multiple regression analysis for the predicting association of TT_3 levels and other clinical factors with NYHA class.

Predictors	$ß$ †	p ‡
Age (years)	0.069	0.365
BMI (kg/m^2)	-0.008	0.915
Total T_3	-0.042	0.603
Total testosterone (nmol/L)	-0.014	0.847
Glomerular filtration rate ($ml/min/1.73m^2$)	-0.314	<0.001 *
Arterial hypertension	0.028	0.751
Diabetes mellitus	0.130	0.080
Hyperlipidaemia	-0.037	0.585
Previous MI	-0.083	0.224
Smoking	-0.062	0.344
Alcohol consumption	-0.130	0.055
Loop diuretic	-0.018	0.823
Spironolactone	0.154	0.031 *
Beta-blocker	-0.035	0.650
Calcium channel blocker	0.040	0.581
ACEI	-0.027	0.697
ARB	0.139	0.052
Digoxin	0.207	0.005 *

* Statistically significant ($p<0.05$).

†, ‡ $ß$ and p-values were obtained from the multiple regression analysis.

TT_3: total triiodothyronine, NYHA: New York Heart Association, HF: heart failure, BMI: body mass index, MI: myocardial infarction, ACEI: angiotensin-converting enzyme inhibitor, ARB: angiotensin II-receptor blocker.

Supplementary Table S3. Multiple regression analysis for the predicting association of TT_3 levels and other clinical factors with HF duration.

Predictors	*ß* †	*p* ‡
Age (years)	0.201	0.008 *
BMI (kg/m^2)	0.161	0.033 *
Total T_3	-0.079	0.325
Total testosterone (nmol/L)	0.238	0.001 *
Glomerular filtration rate (ml/min/1.73m^2)	0.131	0.101
Arterial hypertension	0.160	0.071
Diabetes mellitus	0.065	0.380
Hyperlipidaemia	-0.104	0.118
Previous MI	-0.048	0.479
Smoking	-0.031	0.641
Alcohol consumption	-0.290	<0.001 *
Loop diuretic	0.100	0.214
Spironolactone	0.109	0.122
Beta-blocker	-0.081	0.292
Calcium channel blocker	0.021	0.774
ACEI	0.031	0.650
ARB	0.285	<0.001 *
Digoxin	0.001	0.989

* Statistically significant ($p<0.05$).

†, ‡ *ß* and *p*-values were obtained from the multiple regression analysis.

TT_3: total triiodothyronine, HF: heart failure, BMI: body mass index, MI: myocardial infarction, ACEI: angiotensin-converting enzyme inhibitor, ARB: angiotensin II-receptor blocker.

Supplementary Table S4. Correlation between echocardiographic parameters of cardiac function, heart failure clinical parameters, and thyroid hormones other than TT_3.

	fT_3 [pmol/L]		fT_4 [pmol/L]		TT_4 [nmol/L]		TSH [mIU/L]	
	r †	*p* ‡	*r*	*p*	*r*	*p*	*r*	*p*
Ejection fraction (%)	-0.201	0.003 *	-0.197	0.004 *	-0.105	0.126	-0.189	0.005 *
Diastolic dysfunction	0.077	0.263	0.040	0.561	-0.026	0.700	0.055	0.421
NT-proBNP [pg/mL]	0.003	0.967	0.049	0.474	0.046	0.503	0.136	0.046 *
NYHA class	-0.066	0.334	-0.045	0.515	0.086	0.208	0.184	0.007 *
HF duration (months)	-0.039	0.566	-0.211	0.002 *	-0.138	0.044 *	-0.150	0.028 *

* Statistically significant ($p<0.05$).

†, ‡ *r* and *p*-values were obtained from the independent samples *t*-test.

The normality of data distribution was tested using the Kolmogorov-Smirnov test.

NT-proBNP: N-terminal pro-type B natriuretic peptide, NYHA: New York Heart Association, HF: heart failure, fT_3: free triiodothyronine, fT_4: free thyroxine, TT_3: total triiodothyronine, TT_4: total thyroxine, TSH: thyroid stimulating hormone.

Supplementary Table S5. Multiple regression analysis for the predicting association of TSH levels and other clinical factors with HF duration.

Predictors	*ß* [†]	*p* [‡]
Age (years)	0.167	0.027 *
BMI (kg/m^2)	0.130	0.066
TSH	-0.179	0.010 *
Total testosterone (nmol/L)	0.224	0.001 *
Glomerular filtration rate ($ml/min/1.73m^2$)	0.053	0.487
Arterial hypertension	0.199	0.022 *
Diabetes mellitus	0.096	0.181
Hyperlipidaemia	-0.132	0.046 *
Previous MI	-0.061	0.353
Smoking	-0.020	0.751
Alcohol consumption	-0.291	<0.001 *
Loop diuretic	0.116	0.144
Spironolactone	0.120	0.023 *
Beta-blocker	-0.141	0.049 *
Calcium channel blocker	-0.016	0.819
ACEI	-0.005	0.946
ARB	0.251	<0.001 *
Digoxin	0.012	0.864

* Statistically significant ($p<0.05$).

[†, ‡] *ß* and *p*-values were obtained from the multiple regression analysis.

TSH: thyroid-stimulating hormone, HF: heart failure, BMI: body mass index, MI: myocardial infarction, ACEI: angiotensin-converting enzyme inhibitor, ARB: angiotensin II-receptor blocker.

Supplementary Table S6. Multiple regression analysis for the predicting association of TSH levels and other clinical factors with left ventricular ejection fraction.

Predictors	*ß* †	*p* ‡
Age (years)	0.124	0.121
BMI (kg/m^2)	-0.006	0.940
TSH	-0.072	0.327
Total testosterone (nmol/L)	0.257	<0.001 *
Glomerular filtration rate (ml/min/1.73m^2)	0.185	0.024 *
Arterial hypertension	0.027	0.770
Diabetes mellitus	-0.165	0.033 *
Hyperlipidaemia	0.141	0.045 *
Previous MI	0.018	0.795
Smoking	0.049	0.481
Alcohol consumption	0.004	0.953
Loop diuretic	0.149	0.079
Spironolactone	-0.135	0.066
Beta-blocker	-0.048	0.533
Calcium channel blocker	0.176	0.023 *
ACEI	0.084	0.252
ARB	0.032	0.824
Digoxin	-0.017	0.941

* Statistically significant (p<0.05).

†, ‡ *ß* and *p*-values were obtained from the multiple regression analysis.

TSH: thyroid-stimulating hormone, HF: heart failure, BMI: body mass index, MI: myocardial infarction, ACEI: angiotensin-converting enzyme inhibitor, ARB: angiotensin II-receptor blocker.

Supplementary Table S7. Multiple regression analysis for the predicting association of TSH levels and other clinical factors with left ventricular diastolic dysfunction.

Predictors	β [†]	p [‡]
Age (years)	0.080	0.313
BMI (kg/m^2)	0.067	0.370
TSH	-0.021	0.770
Total testosterone (nmol/L)	-0.279	<0.001 [*]
Glomerular filtration rate (ml/min/1.73m^2)	-0.148	0.070
Arterial hypertension	0.066	0.470
Diabetes mellitus	0.164	0.032 [*]
Hyperlipidaemia	-0.089	0.203
Previous MI	-0.109	0.119
Smoking	0.011	0.876
Alcohol consumption	0.059	0.403
Loop diuretic	-0.219	0.009 [*]
Spironolactone	0.176	0.016 [*]
Beta-blocker	-0.128	0.091
Calcium channel blocker	-0.240	0.002 [*]
ACEI	0.183	0.012 [*]
ARB	0.106	0.160
Digoxin	0.006	0.935

[*] Statistically significant (p<0.05).

[†, ‡] β and p-values were obtained from the multiple regression analysis.

TSH: thyroid-stimulating hormone, HF: heart failure, BMI: body mass index, MI: myocardial infarction, ACEI: angiotensin-converting enzyme inhibitor, ARB: angiotensin II-receptor blocker.

Supplementary Table S8. Multiple regression analysis for the predicting association of TSH levels and other clinical factors with NT-proBNP levels.

Predictors	*ß* †	*p* ‡
Age (years)	-0.124	0.039 *
BMI (kg/m^2)	-0.088	0.115
TSH	-0.077	0.162
Total testosterone (nmol/L)	-0.321	<0.001 *
Glomerular filtration rate ($ml/min/1.73m^2$)	-0.569	<0.001 *
Arterial hypertension	0.061	0.375
Diabetes mellitus	0.019	0.745
Hyperlipidaemia	0.018	0.733
Previous MI	-0.137	0.010 *
Smoking	-0.056	0.280
Alcohol consumption	0.072	0.175
Loop diuretic	0.062	0.324
Spironolactone	0.255	<0.001 *
Beta-blocker	-0.020	0.723
Calcium channel blocker	-0.094	0.102
ACEI	-0.065	0.238
ARB	-0.263	<0.001 *
Digoxin	-0.051	0.363

* Statistically significant ($p<0.05$).

†, ‡ *ß* and *p*-values were obtained from the multiple regression analysis.

TSH: thyroid-stimulating hormone, NT-proBNP: N-terminal pro-brain natriuretic peptide, HF: heart failure, BMI: body mass index, MI: myocardial infarction, ACEI: angiotensin-converting enzyme inhibitor, ARB: angiotensin II-receptor blocker.

Supplementary Table S9. Multiple regression analysis for the predicting association of TSH levels and other clinical factors with NYHA class.

Predictors	*ß* †	*p* ‡
Age (years)	0.080	0.295
BMI (kg/m^2)	-0.017	0.816
TSH	0.090	0.198
Total testosterone (nmol/L)	-0.026	0.693
Glomerular filtration rate (ml/min/1.73m^2)	-0.302	<0.001 *
Arterial hypertension	0.020	0.817
Diabetes mellitus	0.128	0.081
Hyperlipidaemia	-0.029	0.660
Previous MI	-0.087	0.198
Smoking	-0.075	0.253
Alcohol consumption	-0.131	0.053
Loop diuretic	-0.026	0.750
Spironolactone	0.147	0.035 *
Beta-blocker	-0.034	0.642
Calcium channel blocker	0.058	0.425
ACEI	-0.017	0.804
ARB	0.152	0.035 *
Digoxin	0.190	0.008 *

* Statistically significant (p<0.05).

†, ‡ *ß* and *p*-values were obtained from the multiple regression analysis.

TSH: thyroid-stimulating hormone, NYHA: New York Heart Association, HF: heart failure, BMI: body mass index, MI: myocardial infarction, ACEI: angiotensin-converting enzyme inhibitor, ARB: angiotensin II-receptor blocker.

Supplementary Table S10. Multiple regression analysis for the predicting association of fT_3 levels and other clinical factors with left ventricular ejection fraction.

Predictors	*ß* †	*p* ‡
Age (years)	0.147	0.059
BMI (kg/m²)	0.011	0.885
Free T_3	-0.197	0.004 *
Total testosterone (nmol/L)	0.272	<0.001 *
Glomerular filtration rate (ml/min/1.73m²)	0.195	0.012 *
Arterial hypertension	0.000	0.999
Diabetes mellitus	-0.156	0.039 *
Hyperlipidaemia	0.097	0.171
Previous MI	0.050	0.473
Smoking	0.038	0.571
Alcohol consumption	0.014	0.838
Loop diuretic	0.096	0.258
Spironolactone	-0.121	0.090
Beta-blocker	-0.002	0.977
Calcium channel blocker	0.205	0.006 *
ACEI	0.115	0.109
ARB	0.066	0.368
Digoxin	-0.026	0.722

* Statistically significant ($p<0.05$).

†, ‡ *ß* and *p*-values were obtained from the multiple regression analysis.

fT_3: free triiodothyronine, HF: heart failure, BMI: body mass index, MI: myocardial infarction, ACEI: angiotensin-converting enzyme inhibitor, ARB: angiotensin II-receptor blocker.

Supplementary Table S11. Multiple regression analysis for the predicting association of fT_4 levels and other clinical factors with left ventricular ejection fraction.

Predictors	*ß* †	*p* ‡
Age (years)	0.145	0.061
BMI (kg/m^2)	0.025	0.731
Free T_4	-0.223	0.001 *
Total testosterone (nmol/L)	0.215	0.002 *
Glomerular filtration rate ($ml/min/1.73m^2$)	0.236	0.003 *
Arterial hypertension	-0.026	0.774
Diabetes mellitus	-0.175	0.019 *
Hyperlipidaemia	0.161	0.018 *
Previous MI	0.063	0.368
Smoking	0.072	0.288
Alcohol consumption	0.035	0.616
Loop diuretic	0.121	0.142
Spironolactone	-0.157	0.026 *
Beta-blocker	-0.005	0.950
Calcium channel blocker	0.224	0.003 *
ACEI	0.102	0.149
ARB	0.049	0.501
Digoxin	-0.002	0.979

* Statistically significant (p<0.05).

†, ‡ *ß* and *p*-values were obtained from the multiple regression analysis.

fT_4: free thyroxine, HF: heart failure, BMI: body mass index, MI: myocardial infarction, ACEI: angiotensin-converting enzyme inhibitor, ARB: angiotensin II-receptor blocker.

Supplementary Table S12. Multiple regression analysis for the predicting association of TT_4 levels and other clinical factors with left ventricular ejection fraction.

Predictors	*ß* †	*p* ‡
Age (years)	0.126	0.110
BMI (kg/m^2)	0.010	0.891
Total T_4	-0.140	0.041 *
Total testosterone (nmol/L)	0.243	0.001 *
Glomerular filtration rate ($ml/min/1.73m^2$)	0.210	0.008 *
Arterial hypertension	-0.006	0.944
Diabetes mellitus	-0.195	0.011 *
Hyperlipidaemia	0.159	0.022 *
Previous MI	0.036	0.608
Smoking	0.048	0.481
Alcohol consumption	0.001	0.986
Loop diuretic	0.131	0.120
Spironolactone	-0.151	0.035 *
Beta-blocker	-0.021	0.782
Calcium channel blocker	0.212	0.005 *
ACEI	0.123	0.094
ARB	0.058	0.435
Digoxin	-0.002	0.975

* Statistically significant ($p<0.05$).

†, ‡ *ß* and *p*-values were obtained from the multiple regression analysis.

TT_4: total thyroxine, HF: heart failure, BMI: body mass index, MI: myocardial infarction, ACEI: angiotensin-converting enzyme inhibitor, ARB: angiotensin II-receptor blocker.

Supplementary Table S13. Multiple regression analysis for the predicting association of fT_3 levels and other clinical factors with left ventricular diastolic dysfunction.

	ß †	*p* ‡
Predictors		
Age (years)	0.080	0.310
BMI (kg/m²)	0.063	0.394
Free T_3	0.065	0.339
Total testosterone (nmol/L)	-0.286	<0.001 *
Glomerular filtration rate (ml/min/1.73m²)	-0.138	0.080
Arterial hypertension	0.068	0.454
Diabetes mellitus	0.156	0.040 *
Hyperlipidaemia	-0.069	0.335
Previous MI	-0.119	0.093
Smoking	0.010	0.886
Alcohol consumption	0.055	0.430
Loop diuretic	-0.206	0.016 *
Spironolactone	0.163	0.024 *
Beta-blocker	-0.135	0.073
Calcium channel blocker	-0.240	0.001 *
ACEI	0.180	0.013 *
ARB	0.102	0.170
Digoxin	0.065	0.339

* Statistically significant ($p<0.05$).

†, ‡ *ß* and *p*-values were obtained from the multiple regression analysis.

fT_3: free triiodothyronine, HF: heart failure, BMI: body mass index, MI: myocardial infarction, ACEI: angiotensin-converting enzyme inhibitor, ARB: angiotensin II-receptor blocker.

Supplementary Table S14. Multiple regression analysis for the predicting association of fT_4 levels and other clinical factors with left ventricular diastolic dysfunction.

	ß †	*p* ‡
Predictors		
Age (years)	0.081	0.300
BMI (kg/m^2)	0.061	0.413
Free T_4	0.052	0.448
Total testosterone (nmol/L)	-0.271	<0.001 *
Glomerular filtration rate (ml/min/1.73m^2)	-0.148	0.061
Arterial hypertension	0.073	0.428
Diabetes mellitus	0.162	0.032 *
Hyperlipidaemia	-0.089	0.198
Previous MI	-0.119	0.095
Smoking	0.002	0.982
Alcohol consumption	0.051	0.467
Loop diuretic	-0.216	0.010 *
Spironolactone	0.174	0.015 *
Beta-blocker	-0.131	0.080
Calcium channel blocker	-0.243	0.001 *
ACEI	0.185	0.011 *
ARB	0.108	0.144
Digoxin	-0.002	0.978

* Statistically significant (p<0.05).

†, ‡ *ß* and *p*-values were obtained from the multiple regression analysis.

fT_4: free thyroxine, HF: heart failure, BMI: body mass index, MI: myocardial infarction, ACEI: angiotensin-converting enzyme inhibitor, ARB: angiotensin II-receptor blocker.

Supplementary Table S15. Multiple regression analysis for the predicting association of TT_4 levels and other clinical factors with left ventricular diastolic dysfunction.

Predictors	$ß$ †	p ‡
Age (years)	0.081	0.301
BMI (kg/m^2)	0.070	0.347
Total T_4	-0.028	0.678
Total testosterone (nmol/L)	-0.282	<0.001 *
Glomerular filtration rate (ml/min/1.73m^2)	-0.141	0.074
Arterial hypertension	0.059	0.524
Diabetes mellitus	0.157	0.041 *
Hyperlipidaemia	-0.084	0.225
Previous MI	-0.105	0.134
Smoking	0.010	0.884
Alcohol consumption	0.058	0.408
Loop diuretic	-0.224	0.008 *
Spironolactone	0.171	0.017 *
Beta-blocker	-0.121	0.105
Calcium channel blocker	-0.231	0.002 *
ACEI	0.192	0.009 *
ARB	0.112	0.133
Digoxin	0.008	0.913

* Statistically significant (p<0.05).

†, ‡ $ß$ and p-values were obtained from the multiple regression analysis.

TT_4: total thyroxine, HF: heart failure, BMI: body mass index, MI: myocardial infarction, ACEI: angiotensin-converting enzyme inhibitor, ARB: angiotensin II-receptor blocker.

Supplementary Table S16. Multiple regression analysis for the predicting association of fT_3 levels and other clinical factors with NT-proBNP levels.

Predictors	*ß* †	*p* ‡
Age (years)	-0.113	0.059
BMI (kg/m^2)	-0.086	0.127
Free T_3	0.017	0.740
Total testosterone (nmol/L)	-0.324	<0.001 *
Glomerular filtration rate ($ml/min/1.73m^2$)	-0.545	<0.001 *
Arterial hypertension	0.051	0.462
Diabetes mellitus	0.009	0.873
Hyperlipidaemia	0.031	0.561
Previous MI	-0.139	0.010 *
Smoking	-0.063	0.222
Alcohol consumption	0.071	0.183
Loop diuretic	0.060	0.355
Spironolactone	0.240	<0.001 *
Beta-blocker	-0.009	0.872
Calcium channel blocker	-0.079	0.162
ACEI	-0.054	0.320
ARB	-0.252	<0.001 *
Digoxin	-0.061	0.280

* Statistically significant ($p<0.05$).

†, ‡ *ß* and *p*-values were obtained from the multiple regression analysis.

fT_3: free triiodothyronine, NT-proBNP: N-terminal pro-brain natriuretic peptide, HF: heart failure, BMI: body mass index, MI: myocardial infarction, ACEI: angiotensin-converting enzyme inhibitor, ARB: angiotensin II-receptor blocker.

Supplementary Table S17. Multiple regression analysis for the predicting association of fT_4 levels and other clinical factors with NT-proBNP levels.

	ß †	*p* ‡
Predictors		
Age (years)	-0.116	0.050
BMI (kg/m^2)	-0.097	0.085
Free T_4	0.095	0.066
Total testosterone (nmol/L)	-0.306	<0.001 *
Glomerular filtration rate (ml/min/1.73m^2)	-0.559	<0.001 *
Arterial hypertension	0.067	0.330
Diabetes mellitus	0.012	0.835
Hyperlipidaemia	0.022	0.675
Previous MI	-0.154	0.004 *
Smoking	-0.076	0.140
Alcohol consumption	0.059	0.271
Loop diuretic	0.065	0.304
Spironolactone	0.247	<0.001 *
Beta-blocker	-0.019	0.734
Calcium channel blocker	-0.092	0.104
ACEI	-0.056	0.303
ARB	-0.252	<0.001 *
Digoxin	-0.071	0.206

* Statistically significant (p<0.05).

†, ‡ *ß* and *p*-values were obtained from the multiple regression analysis.

fT_4: free thyroxine, NT-proBNP: N-terminal pro-brain natriuretic peptide, HF: heart failure, BMI: body mass index, MI: myocardial infarction, ACEI: angiotensin-converting enzyme inhibitor, ARB: angiotensin II-receptor blocker.

Supplementary Table S18. Multiple regression analysis for the predicting association of TT_4 levels and other clinical factors with NT-proBNP levels.

Predictors	*ß* †	*p* ‡
Age (years)	-0.108	0.070
BMI (kg/m^2)	-0.090	0.110
Total T_4	0.057	0.267
Total testosterone (nmol/L)	-0.318	<0.001 *
Glomerular filtration rate (ml/min/1.73m^2)	-0.548	<0.001 *
Arterial hypertension	0.059	0.397
Diabetes mellitus	0.020	0.728
Hyperlipidaemia	0.023	0.662
Previous MI	-0.143	0.008 *
Smoking	-0.066	0.200
Alcohol consumption	0.073	0.170
Loop diuretic	0.061	0.339
Spironolactone	0.244	<0.001 *
Beta-blocker	-0.012	0.832
Calcium channel blocker	-0.087	0.129
ACEI	-0.064	0.248
ARB	-0.255	<0.001 *
Digoxin	-0.070	0.216

* Statistically significant (p<0.05).

†, ‡ *ß* and *p*-values were obtained from the multiple regression analysis.

TT_4: total thyroxine, NT-proBNP: N-terminal pro-brain natriuretic peptide, HF: heart failure, BMI: body mass index, MI: myocardial infarction, ACEI: angiotensin-converting enzyme inhibitor, ARB: angiotensin II-receptor blocker.

Supplementary Table S19. Multiple regression analysis for the predicting association of fT_4 levels and other clinical factors with HF duration.

Predictors	*ß* †	*p* ‡
Age (years)	0.201	0.007 *
BMI (kg/m^2)	0.155	0.029 *
Free T_4	-0.143	0.029 *
Total testosterone (nmol/L)	0.193	0.005 *
Glomerular filtration rate (ml/min/1.73m^2)	0.125	0.097
Arterial hypertension	0.145	0.098
Diabetes mellitus	0.076	0.292
Hyperlipidaemia	-0.103	0.117
Previous MI	-0.030	0.656
Smoking	-0.019	0.776
Alcohol consumption	-0.271	<0.001 *
Loop diuretic	0.086	0.278
Spironolactone	0.120	0.078
Beta-blocker	-0.090	0.209
Calcium channel blocker	0.042	0.559
ACEI	0.028	0.685
ARB	0.284	<0.001 *
Digoxin	0.005	0.941

* Statistically significant ($p<0.05$).

†, ‡ *ß* and *p*-values were obtained from the multiple regression analysis.

fT_4: free thyroxine, HF: heart failure, BMI: body mass index, MI: myocardial infarction, ACEI: angiotensin-converting enzyme inhibitor, ARB: angiotensin II-receptor blocker.

Supplementary Table S20. Multiple regression analysis for the predicting association of TT_4 levels and other clinical factors with NYHA class.

Predictors	*ß* †	*p* ‡
Age (years)	0.075	0.318
BMI (kg/m²)	-0.033	0.647
Total T_4	0.132	0.043 *
Total testosterone (nmol/L)	-0.013	0.848
Glomerular filtration rate (ml/min/1.73m²)	-0.331	<0.001 *
Arterial hypertension	0.056	0.526
Diabetes mellitus	0.159	0.030 *
Hyperlipidaemia	-0.049	0.456
Previous MI	-0.104	0.123
Smoking	-0.072	0.265
Alcohol consumption	-0.128	0.057
Loop diuretic	-0.006	0.938
Spironolactone	0.166	0.015 *
Beta-blocker	-0.063	0.377
Calcium channel blocker	0.020	0.779
ACEI	-0.057	0.412
ARB	0.124	0.081
Digoxin	0.180	0.013 *

* Statistically significant ($p<0.05$).

†, ‡ *ß* and *p*-values were obtained from the multiple regression analysis.

TT_4: total thyroxine, NYHA: New York Heart Association, HF: heart failure, BMI: body mass index, MI: myocardial infarction, ACEI: angiotensin-converting enzyme inhibitor, ARB: angiotensin II-receptor blocker.

Supplementary Table S21. Multiple regression analysis for the predicting association of fT_3 levels and other clinical factors with NYHA class.

Predictors	*ß* †	*p* ‡
Age (years)	0.070	0.352
BMI (kg/m^2)	-0.015	0.833
Free T_3	-0.085	0.199
Total testosterone (nmol/L)	-0.016	0.811
Glomerular filtration rate (ml/min/1.73m^2)	-0.333	<0.001 *
Arterial hypertension	0.028	0.753
Diabetes mellitus	0.144	0.049
Hyperlipidaemia	-0.062	0.363
Previous MI	-0.075	0.268
Smoking	-0.068	0.300
Alcohol consumption	-0.126	0.062
Loop diuretic	-0.038	0.642
Spironolactone	0.174	0.013 *
Beta-blocker	-0.036	0.618
Calcium channel blocker	0.046	0.525
ACEI	-0.023	0.739
ARB	0.147	0.041 *
Digoxin	0.201	0.005 *

* Statistically significant ($p<0.05$).

†, ‡ *ß* and *p*-values were obtained from the multiple regression analysis.

fT_3: free triiodothyronine, NYHA: New York Heart Association, HF: heart failure, BMI: body mass index, MI: myocardial infarction, ACEI: angiotensin-converting enzyme inhibitor, ARB: angiotensin II-receptor blocker.

Supplementary Table S22. Multiple regression analysis for the predicting association of fT_3 levels and other clinical factors with HF duration.

Predictors	*ß* †	*p* ‡
Age (years)	0.199	0.008 *
BMI (kg/m^2)	0.143	0.046 *
Free T_3	-0.076	0.245
Total testosterone (nmol/L)	0.225	0.001 *
Glomerular filtration rate (ml/min/1.73m^2)	0.101	0.180
Arterial hypertension	0.166	0.059
Diabetes mellitus	0.084	0.248
Hyperlipidaemia	-0.131	0.056
Previous MI	-0.046	0.495
Smoking	-0.039	0.545
Alcohol consumption	-0.287	<0.001 *
Loop diuretic	0.082	0.315
Spironolactone	0.136	0.049 *
Beta-blocker	-0.096	0.182
Calcium channel blocker	0.026	0.717
ACEI	0.031	0.656
ARB	0.289	<0.001 *
Digoxin	-0.010	0.886

* Statistically significant (p<0.05).

†, ‡ *ß* and *p*-values were obtained from the multiple regression analysis.

fT_3: free triiodothyronine, HF: heart failure, BMI: body mass index, MI: myocardial infarction, ACEI: angiotensin-converting enzyme inhibitor, ARB: angiotensin II-receptor blocker.

Supplementary Table S23. Multiple regression analysis for the predicting association of fT_4 levels and other clinical factors with NYHA class.

	β [†]	p [‡]
Predictors		
Age (years)	0.065	0.388
BMI (kg/m^2)	-0.021	0.768
Free T_4	0.004	0.947
Total testosterone (nmol/L)	-0.023	0.737
Glomerular filtration rate (ml/min/1.73m^2)	-0.328	<0.001 *
Arterial hypertension	0.035	0.692
Diabetes mellitus	0.137	0.061
Hyperlipidaemia	-0.040	0.547
Previous MI	-0.089	0.195
Smoking	-0.067	0.312
Alcohol consumption	-0.131	0.055
Loop diuretic	-0.017	0.830
Spironolactone	0.163	0.019 *
Beta-blocker	-0.050	0.485
Calcium channel blocker	0.039	0.591
ACEI	-0.032	0.648
ARB	0.137	0.056
Digoxin	0.201	0.006 *

[*] Statistically significant ($p<0.05$).

[†, ‡] β and p-values were obtained from the multiple regression analysis.

fT_4: free thyroxine, NYHA: New York Heart Association, HF: heart failure, BMI: body mass index, MI: myocardial infarction, ACEI: angiotensin-converting enzyme inhibitor, ARB: angiotensin II-receptor blocker.

Supplementary Table S24. Multiple regression analysis for the predicting association of TT_4 levels and other clinical factors with HF duration.

Predictors	*ß* †	*p* ‡
Age (years)	0.189	0.012 *
BMI (kg/m^2)	0.145	0.043 *
Total T_4	-0.085	0.190
Total testosterone (nmol/L)	0.211	0.002 *
Glomerular filtration rate ($ml/min/1.73m^2$)	0.107	0.153
Arterial hypertension	0.158	0.073
Diabetes mellitus	0.063	0.386
Hyperlipidaemia	-0.105	0.115
Previous MI	-0.048	0.476
Smoking	-0.034	0.600
Alcohol consumption	-0.293	<0.001 *
Loop diuretic	0.093	0.248
Spironolactone	0.124	0.070
Beta-blocker	-0.100	0.161
Calcium channel blocker	0.033	0.645
ACEI	0.040	0.567
ARB	0.289	<0.001 *
Digoxin	0.004	0.953

* Statistically significant ($p<0.05$).

†, ‡ *ß* and *p*-values were obtained from the multiple regression analysis.

TT_4: total thyroxine, HF: heart failure, BMI: body mass index, MI: myocardial infarction, ACEI: angiotensin-converting enzyme inhibitor, ARB: angiotensin II-receptor blocker.
